# Assessing Soil Organic Matter Content in a Coal Mining Area through Spectral Variables of Different Numbers of Dimensions

**DOI:** 10.3390/s20061795

**Published:** 2020-03-24

**Authors:** Chuanmei Zhu, Zipeng Zhang, Hongwei Wang, Jingzhe Wang, Shengtian Yang

**Affiliations:** 1 Key Laboratory of Oasis Ecology, Xinjiang University, Urumqi 830046, China; aspiration818@163.com (C.Z.); zp_zhang@stu.xju.edu.cn (Z.Z.); 2 Key Laboratory of Smart City and Environment Modelling of Higher Education Institute, College of Resources and Environment Sciences, Xinjiang University, Urumqi 830046, China; 3 Key Laboratory for Geo-Environmental Monitoring of Coastal Zone of the Ministry of Natural Resources & Guangdong Key Laboratory of Urban Informatics & Shenzhen Key Laboratory of Spatial Smart Sensing and Services, Shenzhen University, Shenzhen 518060, China; jingzhewang@szu.edu.cn; 4 College of Water Sciences, Beijing Normal University, Beijing Key Laboratory of Urban Hydrological Cycle and Sponge City Technology, Beijing 100875, China; yangshengtian@bnu.edu.cn

**Keywords:** visible and near-infrared spectroscopy, principal component analysis, optimal band combination algorithm, random forest, three-dimensional slice map

## Abstract

Soil organic matter (SOM) is a crucial indicator for evaluating soil quality and an important component of soil carbon pools, which play a vital role in terrestrial ecosystems. Rapid, non-destructive and accurate monitoring of SOM content is of great significance for the environmental management and ecological restoration of mining areas. Visible-near-infrared (Vis-NIR) spectroscopy has proven its applicability in estimating SOM over the years. In this study, 168 soil samples were collected from the Zhundong coal field of Xinjiang Province, Northwest China. The SOM content (g kg^−1^) was determined by the potassium dichromate external heating method and the soil reflectance spectra were measured by the spectrometer. Two spectral feature extraction strategies, namely, principal component analysis (PCA) and the optimal band combination algorithm, were introduced to choose spectral variables. Linear models and random forests (RF) were used for predictive models. The coefficient of determination (*R*^2^), root mean square error (RMSE), and the ratio of the performance to the interquartile distance (RPIQ) were used to evaluate the predictive performance of the model. The results indicated that the variables (2DI and 3DI) derived from the optimal band combination algorithm outperformed the PCA variables (1DV) regardless of whether linear or RF models were used. An inherent gap exists between 2DI and 3DI, and the performance of 2DI is significantly poorer than that of 3DI. The accuracy of the prediction model increases with the increasing number of spectral variable dimensions (in the following order: 1DV < 2DI < 3DI). This study proves that the 3DI is the first choice for the optimal band combination algorithm to derive sensitive parameters related to SOM in the coal mining area. Furthermore, the optimal band combination algorithm can be applied to hyperspectral or multispectral images and to convert the spectral response into image pixels, which may be helpful for a soil property spatial distribution map.

## 1. Introduction

The mining and processing of mineral resources have shown no shortage of economic benefits. However, coal mining will disturb the soil layer, destroy vegetation and cause the soil to lose its utilization value [1]. These issues have posed a serious threat to the sustainable development of land resources and the ecological environment [2]. The area of land damaged by coal mining every year at the global scale is estimated to exceed 12.5 hm^2^ [3,4]. In China, large open-pit coal mines are mainly concentrated in ecologically fragile zones under drought and semi-drought [5]. The self-repairing ability of the soil in this region is relatively poor, and the ecological sensitivity is relatively strong [6]. The long-term development of mineral resources has resulted in serious environmental problems and disasters. Soil is the foundation of many ecological processes (e.g., nutrient cycling, water balance, litter decomposition, etc.) in terrestrial ecosystems [7]. Soil organic matter (SOM) is a crucial indicator for evaluating soil quality and an important component of soil carbon pools, which play a vital role in terrestrial ecosystems [8,9,10]. Therefore, the rapid, repetitive and accurate monitoring of SOM content is of great significance for the environmental management and ecological restoration of mining areas [11,12]. 

With the advancement of science and technology in recent decades, visible-near-infrared (Vis-NIR) spectroscopy has become practical and affordable and has gradually begun to replace or assist experimental analysis [13,14,15]. SOM has obvious spectral characteristics and is the main factor affecting spectral deformation [1,16]. In the Vis region, it is dominated by the electron transition in the chromophore, while in the NIR, it is manifested as the overtones and combined absorption of the molecular vibrations of N-H, C-O, C-H, and other bonds [7].

Hyperspectral data are characterized by a large amount of data and multicollinearity and are usually composed of three types of spectral information: valid information, redundant information, and invalid information [10]. Using full-band modeling will be interfered with by noise and redundant information, which will lead to unsatisfactory results. However, there is increasing evidence that choosing the informative spectral variable can reduce the model’s complexity and improve the prediction performance [17,18,19]. Principal component analysis (PCA) is a common method to reduce the dimensionality of spectral data in obtaining effective variables [7,9]. The optimal band combination algorithm has the advantage of enhancing the correlation between the specific properties and spectral characteristics of a target, minimizing the influence of other irrelevant variables, and is widely applied in soil evaluation [18,20,21,22]. Previous studies have mostly explored the performance of indices in the form of two bands [1,23,24], while the potential of the three-band index (TBI) form has remained unknown.

Random forest (RF) is a machine learning method widely used in the field of classification and regression in recent years [25]. Multiple soil properties have been linked to the Vis-NIR spectrum through this algorithm, such as soil organic carbon (SOC) [26], soil cadmium (Cd) [27], forage phosphorus (P) [28] and soil pH [29]. Numerous research results indicate that RF provides better prediction results than the classical partial least-squares regression (PLSR) [29,30,31]. Furthermore, compared to other machine learning technologies such as artificial neural networks (ANN) and support vector machine (SVM), the RF model can provide the relative importance of each predictor, which makes the results of the model highly interpretable [27,32]. 

The core of this study is to compute sensitive spectral variables by increasing the dimension of the combination of spectral variables, so a series of spectral features (i.e., valid features, redundant features, and invalid features) are generated. Then, we reduce the dimension of the variables and choose the optimal spectral parameters to predict the SOM content in the mining area by the RF model.

The purpose of this study attempts to answer the following questions:

(i) Are there differences in prediction performances based on different feature selection methods (PCA and optimal band combination algorithm)?
(ii) When the dimension of spectral parameters increases, what effect does it have on the predictive performance of SOM content?
(iii) Can an acceptable prediction result be obtained from a single spectral parameter alone?
(iv) Can the selected optimal parameters be interpreted in terms of known soil properties or functional groups?

## 2. Materials and Methods

### 2.1. Study Areas and Soil Sampling

**Figure 1:**
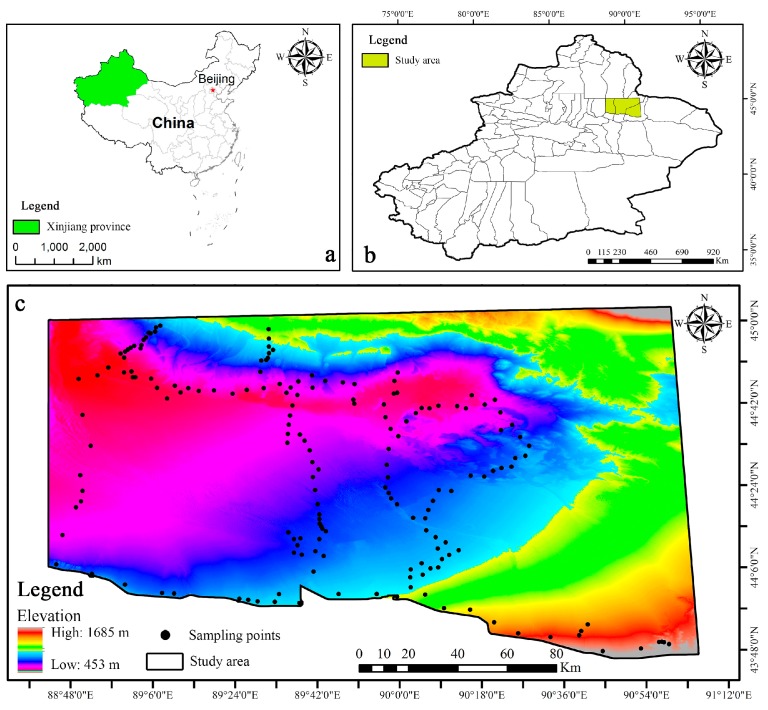
Geographical location of Xinjiang Province (**a**) and study area (**b**), and spatial distribution of sampling points (**c**).

The research area is the Zhundong coalfield, which is located southeast of the Junggar basin of China (43°45′-45°00′ N and 88°45′-91°10′ E) (Figure 1b). The coalfield covers an area of approximately 13,000 square kilometers, and the predicted coal reserves reach 390 billion tons. It is the largest integrated coalfield in the world and is known as “the granary of China’s industry”. The study area ranges in elevation and slope from 453 m to 1685 m and from 0° to 89°, respectively (Figure 1b). The site is in an extremely dry continental climate with average annual precipitation and temperatures from 140 to 183 mm and 5.3° to 7.3°, respectively [33]. According to the World Reference Base soil classification and soil taxonomy [34], the common soil types of the study region are arenosols, solvents, and gypsisols. The soil parent material is Quaternary alluvial deposits, and the surface vegetation is sparse. The mainland use and land cover (LU/LC) types in the Zhundong coalfield are bare lands, grassland and cropland [35]. Since the start of the Zhundong coal mine in 2006, heavy industrial and mining activities have caused ecological imbalances and serious environmental pollution, and the soil properties may be changing.

A field survey was conducted in mid-June 2014, and we collected a total of 168 soil samples (Figure 1c); during the collection period, no extreme weather (such as heavy rain or strong winds) occurred. The main terrain of the study area is hilly, so the main route designed for the experiment was along the roads. A relatively flat area away from the road (>300 m) was selected as the sampling site to ensure the safety of vehicles and personnel. At each sampling point, topsoil samples (depth range from 0 to 20 cm) from five sub-samples within an area 10 m in diameter were collected with a wooden shovel and mixed into composite samples (approximately 1.5 kg). The composite samples were immediately loaded into a labeled waterproof ziplock bag, and the coordinates, elevation and vegetation coverage of the samples were recorded using a handheld GPS and vegetation coverage meter. The samples were brought back to the laboratory for indoor air drying for 2 weeks (room temperature ranging from 26 to 28 °C), non-soil materials (e.g., gravel, plant roots, and other materials) were carefully removed, and the samples were then gently crushed with an agate mortar and passed through a 1.5 mm sieve to reduce the impact of particle size. The SOM content (g kg^−1^) was determined by the potassium dichromate external heating method [36].

### 2.2. Vis-NIR Spectroscopy Measurement and Pre-Processing

The ASD FieldSpec^®^ Pro FR spectrometer (Malvern Panalytical Ltd, Malvern, UK) was used for acquisition of the soil reflectance spectra in the wavelength range of 350–2500 nm (output spectral interval of 1 nm). The spectral acquisition process was as follows: in a dark room, a 50 W halogen lamp (Malvern Panalytical Ltd, Malvern, UK) with an incident angle of 45° was mounted 60 cm above the center of the sample to provide illumination. The soil sample was placed in a pre-cleaned petri dish (1.0 cm in height × 3.6 cm in diameter), and a spatula was used to make its surface smooth. Each sample was repeatedly scanned 12 times over the central area of the sample, and the average value was taken as the reference spectrum after removing the abnormal spectrum. For each sample measurement, we used a square white BaSO_4_ panel (with 99% reflectance) to calibrate the spectrometer.

Overall, the wavelength on the fringe (the first 50 nm and the last 100 nm) of the spectrometers had a relatively low signal-to-noise ratio [11]. Therefore, we retained only the wavelengths from 400 nm to 2400 nm. The total number of spectral bands was reduced from 2001 to 401 to improve the calculation efficiency and eliminate the redundant information using a Gaussian filter. The Savitzky–Golay filter (with a polynomial order of two and window size of 11) was used to further reduce the signal noise of the spectrum [37]. Continuum removal (CR) was used to extract spectral characteristics to facilitate spectral interpretation [38]. All pre-processing in this study was carried out in MATLAB version R2018b (MathWorks, Natick, MA, USA).

### 2.3. Vis-NIR Spectral Feature Extraction

In this study, PCA and the optimal band combination algorithm were used to conduct spectral information extraction. PCA is a statistical analysis method widely used in spectral data feature extraction, compression and dimensionality reduction [39]. Through orthogonal transformation, it can convert the original spectral dataset into a set of uncorrelated principal components (PCs) under the premise of retaining the original data information as much as possible [7]. The number of PCs used for identifying spectral data was selected based on the cumulative contribution rate, and components with a cumulative contribution rate greater than 95% were chosen. The extracted PCs are defined as one-dimensional variables (1DVs) and divided into calibration and validation datasets (described in the next section) for subsequent analysis and model input. To further explore the spectral variation of the soil samples, we performed PCA on the CR spectra of the entire dataset (*n* = 168).

The optimal band combination algorithm can extract the most sensitive spectral information and simplify the influence of unrelated bands [17]. According to previous research [17,27], we used the following five two-band index forms to explore the relationship between SOM content and spectral variables. The mathematical expression is characterized by Equations (1)−(5):

SI(*R_i_*, *R_j_*) ^a^ = (*R_i_* + *R_j_*) (1)

DI(*R_i_*, *R_j_*) ^b^ = (*R_i_* − *R_j_*) (2)

PI(*R_i_*, *R_j_*) ^c^ = (*R_i_* × *R_j_*) (3)

RI(*R_i_*, *R_j_*) ^d^ = (*R_i/_R_j_*) (4)

NDI(*R_i_*, *R_j_*) ^e^ = (*R_i_* − *R_j_*)/(*R_i_* + *R_j_*) (5)

**Note:** ^a^ sum index; ^b^ difference index; ^c^ product index; ^d^ ratio index; ^e^ normalized difference index.

Zhang, et al. [40] reported that adding a third band for a specific sensitive region to the two-band spectral index can significantly improve the estimation accuracy of both and enhance the anti-interference ability. Therefore, we derived five types of three-band indices. The mathematical expressions are as follows (Equations (6)−(10)).

TBI1(*R_i_*, *R_j_*, *R_k_*) ^a^ = *R_i/_*(*R_j_* + *R_k_*) (6)

TBI2(*R_i_*, *R_j_*, *R_k_*) ^b^ = (*R_i_ + R_j_*)/*R_k_* (7)

TBI3(*R_i_*, *R_j_*, *R_k_*) ^c^ = (*R_i_* − *R_j_*)/(*R_j_* − *R_k_*) (8)

TBI4(*R_i_*, *R_j_*, *R_k_*) ^d^ = (*R_i_* − *R_j_*)/(*R_i_* + *R_j_* − 2*R_k_*) (9)

TBI5(*R_i_*, *R_j_*, *R_k_*) ^e^ = *R_i_* + *R_j_* − 2*R_k_* (10)

**Note:** ^a^ three-band index 1; ^b ^ three-band index 2; ^c^ three-band index 3; ^d^ three-band index 4; ^e^ three-band index 5.

Where *R_i_*, *R_j_*, and *R_k_* are the reflectance values at the range of 400−2400 nm, and *R_i_* ≠ *R_j_* ≠ *R_k_*, respectively. The feature extraction procedures of the optimal band combination algorithm involved three steps: (1) for each index form, the Pearson correlation (*r*) between all possible band combinations (in Equations (1)−(5) and (6)−(10) the band combinations were 401^2^ and 401^3^, respectively) and SOM content was traversed; (2) for each index form (in Equations (1)−(10)], the |*r*| values were sorted in ascending order to preserve the top 1% of the band combinations); (3) finally, the optimal band combinations were then selected according to the minimum *r* error between the calibration and validation data sets. The correlation graphs for the contour and slices were used to visualize the optimal band combinations and sensitive spectral response regions. We designed a computer program based on MATLAB version R2018b (MathWorks, Natick, MA, USA) to assist with the calculations and analyses. The optimal band combinations in Equations (1)−(5) and Equations (6)–(10) are denoted as the two-dimensional spectral index form (2DI) and three-dimensional spectral index form (3DI), respectively.

### 2.4. Dataset Division and Modeling Strategy

The 168 samples were sorted in ascending order of SOM content. Then, we placed one of the three samples into the validation set and the other two samples into the calibration set. This procedure led to a calibration set and a validation set with 112 and 56 samples, respectively.

Random forests (RF) are the product of ensemble learning and are currently popular in the fields of classification and regression [25]. Specifically, an RF combines two powerful statistical techniques: boosting and classification and regression tree (CART). The boosting algorithm implements the modeling process by iteratively extracting trees and then returning them to the model [41]. The regression tree performs binary splitting of each resulting section to obtain the split-point that achieves the best model fit, and the combination of regression trees can yield more accurate and stable predictions. Compared with other data mining algorithms, the RF model has better interpretability because it can output the importance of features, which can assist us in evaluating and screening important features [31]. RF modeling was implemented with the package “Regression Tree Ensembles” version 0.02 based on MATLAB version R2018b (MathWorks, Natick, MA, USA).

The fitting of an RF model is controlled by the specification of three parameters: the number of trees (*n_trees_*), the number of variables per tree (*m_try_*), and the minimum number of terminal nodes (*nodesize*). The default value of *n_trees_* (500) has proven to be insufficient to produce stable results [42]. Therefore, we choose an RF model with a *n_trees_* value of 1000. For *nodesize*, we use the default values in the package. For the optimization of *m_try_* value, it ranged from one to five and from one to 10, both were tested at the interval of one for the two-variable combinations (mentioned in the next Section 2.5). 

### 2.5. Statistical Analysis and Flow Chart

Analysis of variance (ANOVA) was used to verify whether significant differences (*p* < 0.05) exist between the calibration and validation sets and between the SOM content and Vis-NIR spectral data [18]. An accuracy assessment was conducted using the coefficient of determination (*R*^2^), root mean square error (RMSE), and the ratio of the performance to the interquartile distance (RPIQ). Bellon-Maurel et al. [43] defined four classes of RPIQ, namely, categories A (RPIQ > 4.05), B (3.37 < RPIQ < 4.05), C (2.70 < RPIQ < 3.37), and D (RPIQ < 2.70), which indicate that a model can excellently predict the property in question, has good predictability, has limited predictability or has poor predictability, respectively. Taking the minimum RMSE in the calibration set as the objective, the optimal *m_try_* value was selected. The confidence intervals and prediction intervals [19] were added to a scatter plot to measure the prediction reliability of the SOM (at the 0.05 confidence level). The confidence interval represents covering the true fitted line with 95% probability, and the prediction interval represents covering all future data points with 95% probability.

**Figure 2:**
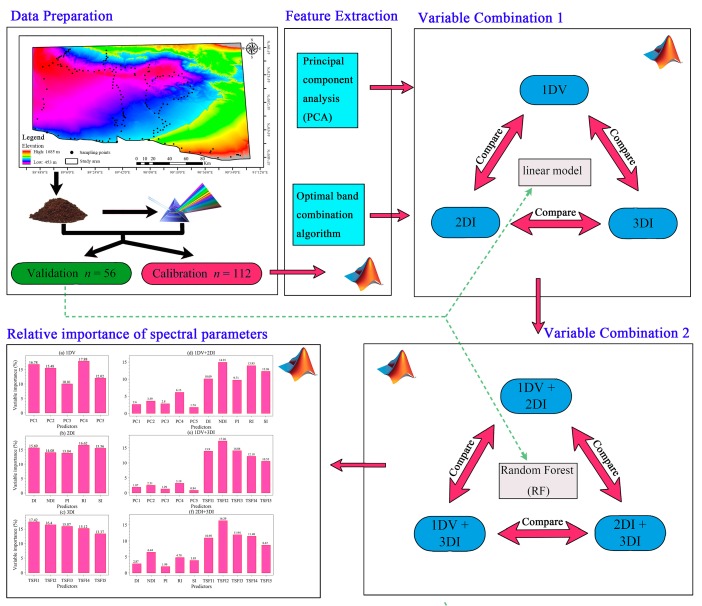
Flow chart of the current work.

To explore the influence and importance of different spectral variable types on the prediction accuracy of the SOM, we first developed estimation models with 1DV, 2DI, or 3DI and then combined them in pairs. Figure 2 presents all the procedures used to predict the SOM content in this study. In Figure 2, the blue arrows indicate the flow of the experiment; the red flat hexagons represent the original data (SOM and Vis-NIR spectral data); the blue and green oval frames correspond to the processed calibration and validation datasets, respectively; and the descriptions in the orange rectangular boxes indicate the experimental operations. The labels 1DV, 2DI and 3DI represent variable combination 1, 1DV+2DI, 1DV+3DI and 2DI+3DI represent variable combination 2.

## 3. Results

### 3.1. Descriptive Statistics of SOM Content

The range of the entire SOM data set was 45.45 g kg^−1^, with a mean value of 7.46 g kg^−1^ (Table 1). The kurtosis and skewness were 5.10 and 2.13, respectively, and both were greater than zero; hence, the distribution of the SOM can be considered offset to the right and steeply distributed. A coefficient of variation (CV) of 117.23% was considered to have high variability, reflecting the differences in soil parent materials and land use. Large soil variability may be beneficial to enhance the predictive accuracy of the calibration model [44]. The pH values ranged from 7.60 to 10.60 (i.e., alkaline to extremely alkaline). The mean value of pH indicates that the topsoil is strongly alkaline. A comparison of the SOM content between the calibration and validation datasets showed no significant difference according to the performance of ANOVA (*p* = 0.99) at the 0.05 significance level.

### 3.2. Spectral Characteristic Analysis

**Figure 3:**
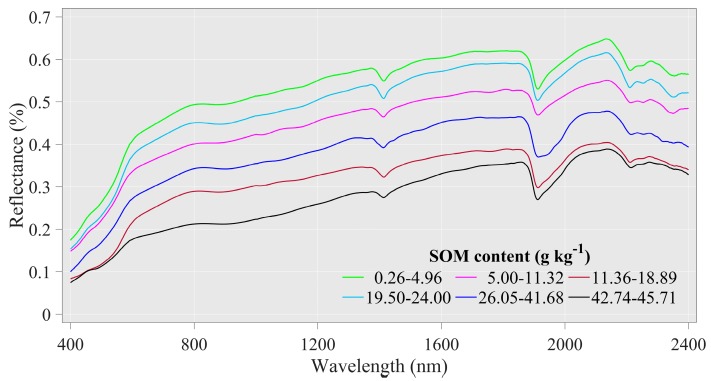
Mean spectral signature of soil organic matter (SOM) content under different classes.

The average spectral reflectance of the different SOM classes is illustrated in Figure 3. In the entire-band range (400–2400 nm), with the increase in SOM, the spectral reflectance decreased gradually; this trend was significant at wavelengths of 400–700 nm, which are mainly associated with minerals that contain iron, as well as the presence of SOM [11,16]. The soil samples from different SOM classes exhibited similar spectral shapes but with variable spectral intensities because the reflectance spectrum in the Vis region is mainly affected by the soil chromophore and the black color of the organic matter itself; therefore, soil with higher SOM is visually brighter than the SOM of light-colored soil [45]. However, in the NIR region, the spectral intensity variation is linked to the double-frequency and combined-frequency absorption of chemical bonds such as N-H, C-H, and C-O [45]. Besides, there are prominent absorption bands at approximately 1400 nm and 1900 nm and weak absorption bands at approximately 2200 nm, similar to previous reports [20,26,46,47,48,49]. These features are caused by the stretch vibration of OH bonds, mainly the absorption of free soil moisture at 1400 and 1900 nm, and by the OH groups in clay lattices at 1400 and 2200 nm [11].

**Figure 4:**
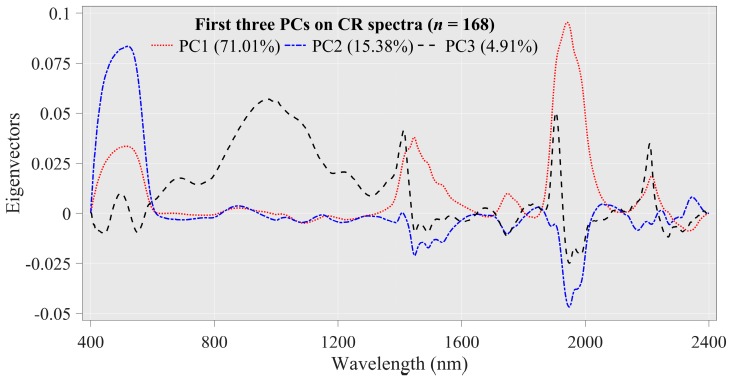
Eigenvector of the first three principal components derived from removal spectra (*n* = 168).

The eigenvectors extracted by PCs were used to identify the obvious absorption characteristics of the SOM content (Figure 4). The cumulative variance contribution rate of the first three PC_S_ was greater than 90%. The eigenvector of PC1 (explaining 71.01% of the total spectral variation) presented three positive peaks within 400–609, 1329–1645 and 1860–2089 nm that can be attributed to the presence of Fe oxides, haematite, O-H, humic acid and smectite [7,50]. In the second PC (explaining 15.38% of the total spectral change), the kurtosis of the positive peak within 400–609 nm was significantly enhanced, while the positive peaks within 1412–1645 and 1860–2089 nm were converted to kurtosis weaker negative peaks [45]. In the third PC (accounting for 4.91% of the total spectral change), the positive peak (in PC1 and PC2) within 400–609 nm was converted into a positive peak between two negative peaks. Moreover, a flat peak within 596–1311 nm and around 1400, 1900 and 2200 nm had both a sharp positive peak and two weak negative peaks, which were associated with the presence of haematite, aromatics, an asymmetric doublet, iron oxides, N-H, C-H, molecular water and clay minerals [45]. However, the specific spectral response of the peaks was sometimes difficult to determine due to the overlapping nature of the hyperspectral data; nevertheless, these preliminary analyses make the Vis-NIR modeling of SOM feasible.

### 3.3. Relationship between SOM Content and Spectral Principal Components

The spectra were compressed using PCA, which was used to summarize the data and visualize the spectral implication of PCs. Overall, in the calibration and validation sets, the correlation coefficients of PCs for different datasets show the same trend; the correlations (*r*) varied from 0.43 (PC4) to –0.37 (PC1) and from 0.31 (PC4) to –0.42 (PC3), respectively (Table 2). The five extracted PCs for the calibration sets are listed in Table 3 for subsequent modeling.

### 3.4. Relationship between SOM Content and Optimal Spectral Indices

**Figure 5:**
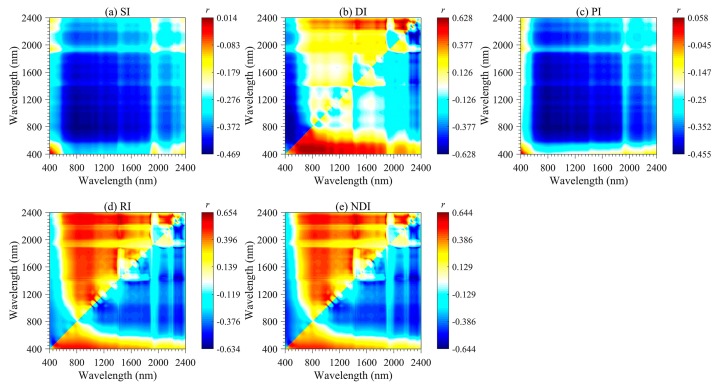
Two-dimensional image maps of correlations (*r*) between SOM and five 2DI (SI, DI, PI, RI, and NDI) forms calculated based on the calibration set (*n* = 112).

According to the correlation coefficient values, the importance region and peak wavelength combination for the SOM could be identified and extracted efficiently (Figure 5). The correlations between DI, RI and NDI and SOM content are symmetrical. The best performing wavelength combinations for RI and NDI appear in the 1400–1500 and 2250–2350 nm regions, and those for DI appear in the 1850–1950 and 2260–2360 nm regions. SI, PI, and SOM content are mainly negatively correlated, and the wavelength located in the 750–850 nm region has a high correlation with the SOM content. Among all 2DV forms, the optimal spectral parameters were provided by RI (*R*_785_, *R*_805_), followed by NDI (*R*_1885_, *R*_2345_), DI (*R*_800_, *R*_790_), SI (*R*_2260_, *R*_1450_) and PI (*R*_1490_, *R*_2340_). The optimal wavelength combinations differed in each 2DV form were extracted for subsequent linear and non-linear regression analyses with the SOM content (Table 3).

**Figure 6:**
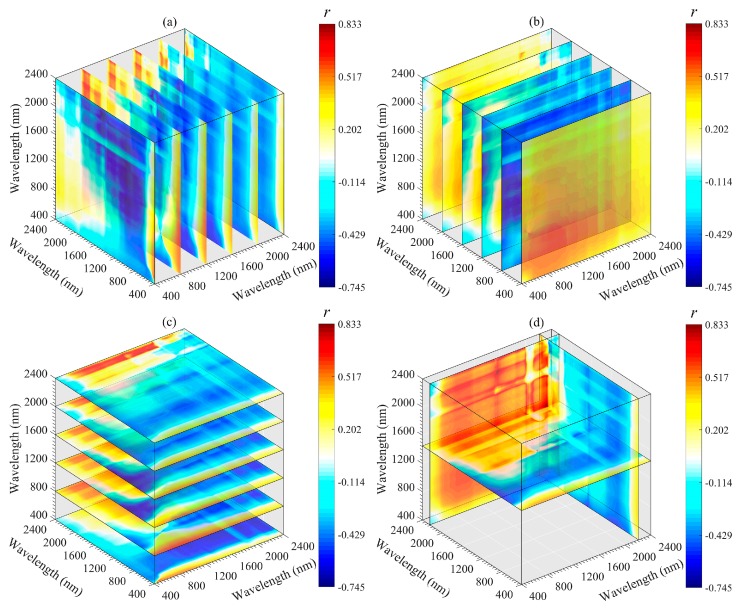
Three-dimensional slice maps of correlations between SOM content and TBI1 based on calibration set (*n* = 112). (**a**–**c**) represent slice maps based on the *x*-axis, *y*-axis, and *z*-axis, respectively, and (**d**) represents the optimal slice map.

**Figure 7:**
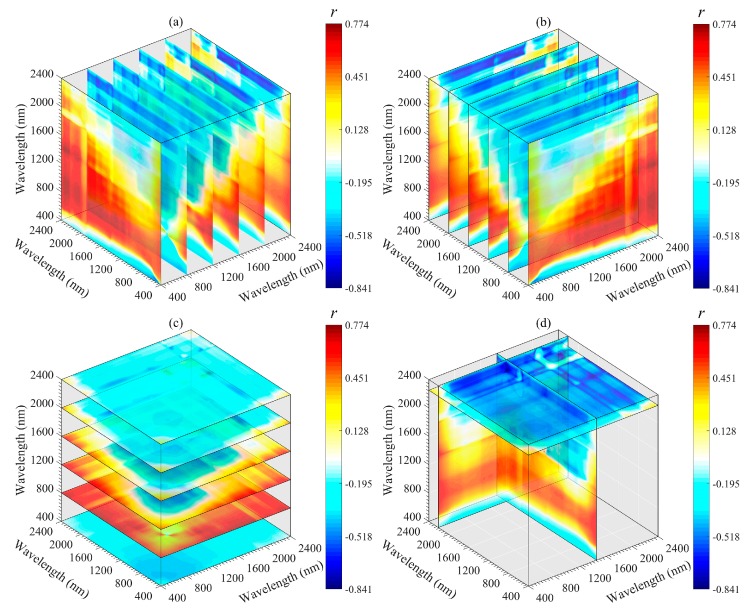
Three-dimensional slice maps of correlations between SOM content and TBI2 based on the calibration set (*n* = 112). (**a**–**c**) represent slice maps based on the *x*-axis, *y*-axis, and *z*-axis, respectively, and (**d**) represents the optimal slice map.

**Figure 8:**
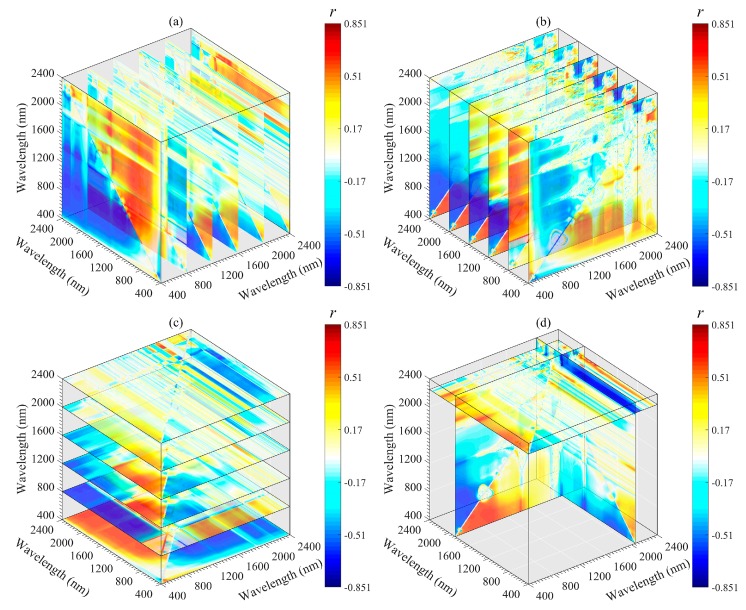
Three-dimensional slice maps of correlations between SOM content and TBI3 based on the calibration set (*n* = 112). (**a**–**c**) represent slice maps based on the *x*-axis, *y*-axis, and *z*-axis, respectively, and (**d**) represents the optimal slice map.

**Figure 9:**
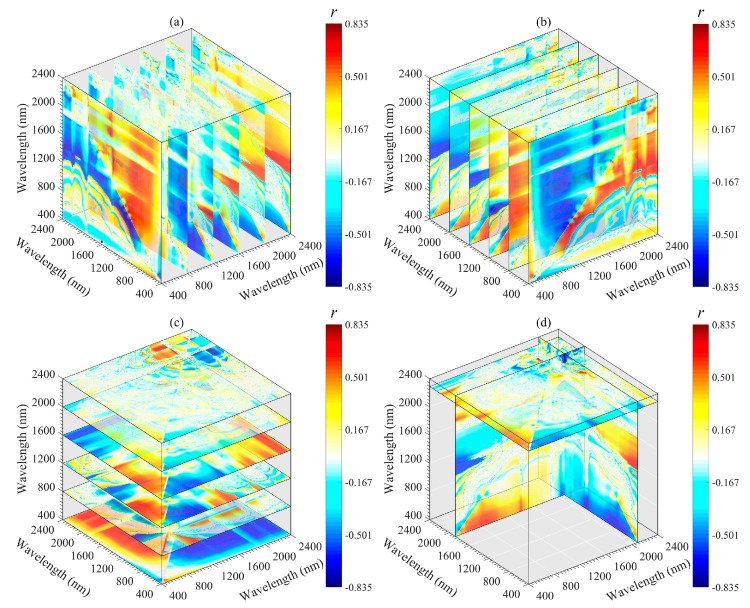
Three-dimensional slice maps of correlations between SOM content and TBI4 based on the calibration set (*n* = 112). (**a**–**c**) represent slice maps based on the *x*-axis, *y*-axis, and *z*-axis, respectively, and (**d**) represents the optimal slice map.

**Figure 10:**
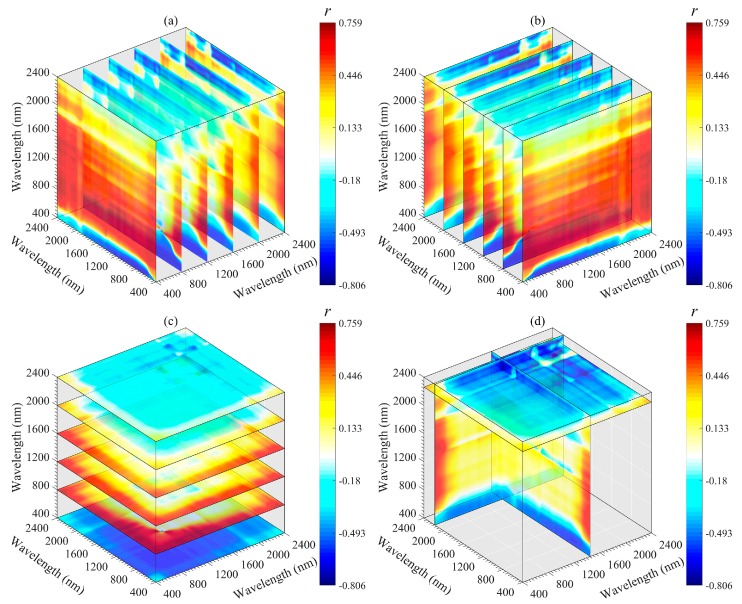
Three-dimensional slice maps of correlations between SOM content and TBI5 based on calibration set (*n* = 112). (**a**–**c**) represent slice maps based on the *x*-axis, *y*-axis, and *z*-axis, respectively, and (**d**) represents the optimal slice map.

The important regions and peak wavelength combinations of the SOM were revealed from different angles (*x*-axis, *y*-axis, *z*-axis, and optimal slice) by using a three-dimensional slice map (Figure 6, Figure 7, Figure 8, Figure 9 and Figure 10). Among all 3DV types, TBI3 (*R*_1890_, *R*_2065_, and *R*_2265_) performed the best in estimating the SOM, and the correlations (*r*) reached a maximum of 0.85 (Figure 8d). The optimal band combinations were inconsistent for each 3DI type (from Figure 6d to Figure 10d). Overall, these peak wavelengths occur in the NIR region, being close to (within ± 10 nm) 1455, 1460, 1465, 1890, 1895, 2065, 2080, 2095, 2200, 2215, 2230, 2250, 2255, 2265, and 2295 nm. Mostly, these wavelengths are related to the OH stretching in the crystal lattice and different organic molecules, such as amides or proteins, clay lattice AL-OH absorption, illite and carbonate organics [11,45]. We extracted them for subsequent linear and non-linear regression analyses with the SOM content (Table 3).

### 3.5. Estimation of SOM with Linear Model and Validation

The 15 potential variables extracted via PCA and the 2DI and 3DI algorithms are summarized in Table 3. Here, the linear model was constructed to quantify the SOM, and the independent validation dataset was utilized to verify the quantitative capabilities of the models. Overall, the five 3DIs outperformed all other 2DI and 1DV models, in terms of both calibration and validation (*R*^2^_c_ ranging from 0.65 to 0.72 and *R*^2^_v_ ranging from 0.64 to 0.70), followed by 2DI (*R*^2^_c_ ranging from 0.33 to 0.48 and *R*^2^_v_ ranging from 0.30 to 0.49) and 1DV (*R*^2^_c_ ranging from 0.01 to 0.19 and *R*^2^_v_ ranging from 0.01 to 0.18). The optimum model yields an RPIQ of 1.50 (< 2.70), indicating that the prediction effect of the model is not reliable. Therefore, the prediction accuracy of a single variable is insufficient, and it is difficult to effectively explain the spatial variation of the SOM in this region.

### 3.6. Estimation of SOM with RF Models and Validation

Using the spectral parameter combination as the model input (see Section 2.5) and RF as the prediction model, six prediction models were established (Table 4). The predictive performance of each model was examined using an independent validation set. The *R*^2^ and RMSE of the calibration and validation sets were then subjected to ANOVA; no statistically significant difference between calibration and verification was found (*p* > 0.05), indicating the stability of the established model (Table 4). Using the spectral variables of a single dimension as model inputs, the 3DI performed better, and the RPIQ reached 3.15 (2.70 < RPIQ < 3.37), indicating that the model accuracy was acceptable, followed by 2DI (RPIQ of 1.97) and 1DV (RPIQ of 1.46). The order of the prediction performance was consistent with Table 3. When the spectral variables of different numbers of dimensions were combined in pairs as model inputs, the prediction accuracy of the combined model was found to be better than that of either of them alone. The 2DI+3DI model achieves excellent estimation (*R*^2^_V_ = 0.93, RMSE_V_ = 2.52 g·kg^−1^ and RPIQ = 4.09); 1DV+3DI yields good outcomes (RPIQ = 3.48); and the 1DV+2DI model had insufficient predictability (RPIQ = 2.48).

**Figure 11:**
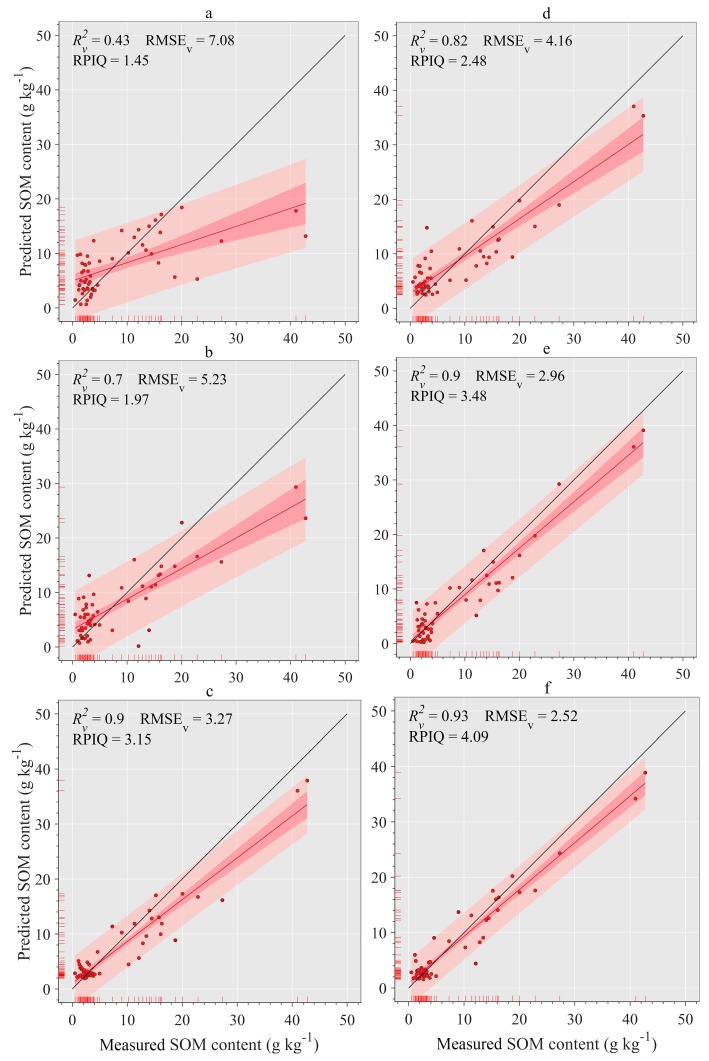
Comparison of measured versus predicted SOM content (*n* = 56) based on the RF model with various modeling strategies. The salmon area (relatively wide area) and crimson area (relatively narrow area) cover the predicted SOM data and its fitting line with 95% probability, respectively. Red and blue lines represent the fitting line and 1:1 line between the measured and predicted SOM content, respectively.

Figure 11 shows the relationship between the measured SOM content and the predicted SOM content for the six modeling strategies in Table 4. In general, we found that the VIS-NIR model is more inclined to overestimate low-value and underestimate high-value SOM content, which is consistent with the results reported by other researchers (Figure 11). The distribution of the predicted versus measured values of the RF model constructed by 1DV deviated from the 1:1 line and had the most outliers compared with the other models (Figure 11a). However, as the dimensions of spectral variables increase, the scatter gradually approaches the 1:1 line in the following order: 1DV < 2DI < 3DI (Figure 11a–c). After a pairwise combination of spectral variables with different numbers of dimensions, the width of the confidence interval and prediction interval was narrower than that of either of them alone (Figure 11d–f). The scatter of the 2DI+3DI model is better distributed along the 1:1 line than those of the other models, with an excellent prediction effect (Figure 11d).

### 3.7. Estimation Mechanism Analysis

**Figure 12:**
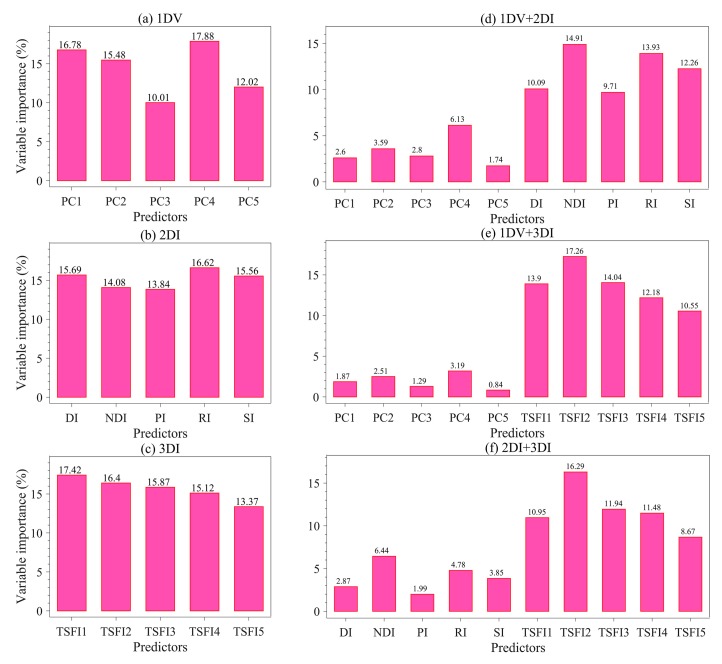
Relative importance (%) of the predictor variable from the RF model in different strategies to predict the SOM content. Each variable of importance was normalized to a 100% scale.

We used the relative importance of the predictor variables derived from the RF model to interpret relationships between the SOM content and different input sources (Figure 12). To the 1DV, 2DI and 3DI models, the importance of single-dimensional spectral variables to the estimation model was not very different; the relative importance varied from 10.01% to 17.88%, 13.84% to 16.62%, and 13.37% to 17.42%, respectively. In the 1DV+2DI model (Figure 12d), NDI contributed the most to the estimation model, followed by RI, SI, DI, and PI. The contribution rate of the principal component variables (from PC1 to PC5) is relatively low (from 1.74% to 6.13%). The relative importance in the 1DV+3DI and 2DI+3DI models was similar to that of the 1DV+2DI model (Figure 12d, Figure 12e). In Figure 12e, the contribution rate of the variables in the 3DI model was significantly higher than that in the 1DV model (Figure 12e), and TSFI2 even reached the largest contribution rate of 17.26%. In contrast, in the 2DI+3DI model (Figure 12f), the importance of the 2DI variables increased compared to the performance of 1DV in Figure 12e.

## 4. Discussion

In electromagnetic theory, SOM is represented as an organic compound containing functional groups with relatively discrete absorption characteristics in the Vis-NIR range [51]. In the visible region (400–760 nm), the transition of the outer electrons from the ground states to high energy states is the primary process of soil energy absorption [11]. In the infrared region, it exhibits the stretching vibration and bending vibration of many organic functional group molecules [45,52]. However, their position usually deviates to some extent from the expected position, because the real molecules do not behave completely harmoniously [11]. Therefore, the interpretation of the soil Vis-NIR spectra becomes more difficult when SOM is present in small amounts in the soil or the research itself depends on the correlation with SOM.

The goal of feature extraction is to minimize errors and exclude unreliable or noisy data to the greatest extent, which is essential to improve the interpretability of the model and/or reduce the complexity of the prediction model [53]. Hong et al. [27] have reported that PCA results in a poorer prediction performance than the optimal band combination algorithm, because the physical meanings of the PCs obtained by PCA are generally not as clear as those of the original spectral variables, thereby worsening the prediction performance. In the present study, the modeling effect of the optimal band combination algorithm was significantly better than PCA (Table 3), which is similar to the above research results. Therefore, we recommend using the optimal band combination algorithm to extract the spectral indices to measure SOM content in the coal mining area.

Whether using the linear model or RF, the results produced by 3DI were more accurate than the results of 2DI, which were indicated by larger *R*^2^ and small RMSE (Table 3 and Table 4). This could be attributed to the fact that that adding a specific sensitive region to the two-band spectral index by adding a third band can generally improve the estimation accuracy of the index and enhance the anti-interference ability [21,22,40,54]. Besides, some studies have proposed constructing a four-band reflection model, but this seems to greatly increase the data dimensions and processing difficulty, resulting in the “curse of dimensionality” [55,56]. Therefore, in the process of designing the spectral index, the complexity and accuracy of the index need to be comprehensively considered, especially for other soil properties. 

Although 2DI+3DI achieved the best prediction effect (*R*^2^_V_ = 0.93, RMSE = 2.52 and RPIQ = 4.09), the contribution rate of 2DI variables was much lower than that of 3DI variables (Figure 12f). Furthermore, some previous studies adopted 2DI as the input predictor and obtained *R*^2^v results from 0.74 to 0.84 for SOM prediction [18,20,23,57]. Therefore, we suggest that the spectral variables of 3DI should be considered first when establishing a spectral model for SOM. Moreover, we still recommend using the optimal band combination algorithm to estimate the SOM because the soil spectrum is a comprehensive reflection of soil attributes, which include not only information about the SOM, but also the known features of OH stretching, soil moisture content, different organic molecules, AL-OH, illites and carbonate organics [45,52]. The bands involved in the optimal spectral indices were close to the absorption features of these components or functional groups. Through these components or functional groups, the SOM content can be retrieved indirectly (Figure 6, Figure 7, Figure 8, Figure 9 and Figure 10).

Although these results show excellent predictive performance, the bandwidth of the spectral indices involved in this study had a 1 nm interval and thus cannot be applied to other ground-based and satellite-based sensors. Therefore, a specific spectral index based on the center band and bandwidth of existing remote sensing sensors must be built by using the optimal band combination algorithm to meet the needs of SOM remote dynamic monitoring. At the same time, since soil samples from different study areas usually exhibit significant spectral differences, it may not be feasible to extrapolate our results to other study areas. However, the technique of using the optimal band combination algorithm to derive a three-band spectral index based on sensitive soil properties can provide a viable solution.

## 5. Conclusions

In this study, two spectral feature extraction strategies (i.e., PCA and the optimal band combination algorithm) were introduced to extract spectral variables. The results indicated that the model performance of the optimal band combination algorithm is better than that of PCA, regardless of whether linear or non-linear models are used. In the variables extracted by the optimal band combination algorithm, the performance of the 2DI is poorer than that of the 3DI. In summary, the accuracy of the prediction model increases with the increasing number of spectral parameter dimensions, which is consistent with our previous hypothesis. Therefore, the three-band index should be the preferred variable for spectral estimation of the SOM. Moreover, compared to the two-dimensional image map, the three-dimensional slice map of the correlation (*r*) displays no shortage of imagination and information. Combined with the method of optimal three-band indices, this study provides useful insights into the assessment of soil nutrition concentrations in other regions.

## Figures and Tables

**Table 1:** Descriptive statistics of soil properties in the study areas.

Soil Properties	Data Set	Min	Mean	Max	STD	IQR	CV (%)	Ske	Kur
SOM (g kg^−1^)	Entire set (*n* = 168)	0.26	7.46	45.71	8.75	10.02	117.23	2.13	5.10
	Calibration set (*n* = 112)	0.26	6.85	45.71	8.38	9.39	122.40	2.24	5.90
	Validation set (*n* = 56)	0.49	7.74	42.74	9.25	10.30	119.53	2.16	5.16
pH	Entire set (*n* = 168)	7.60	9.11	10.60	0.90	1.60	9.91	0.04	1.67

Standard deviation (SD); the interquartile range of the data samples (IQR); coefficient of variation (CV); skewness (Ske); kurtosis (Kur).

**Table 2:** Correlations (*r*) between SOM content and principal component analysis (PCA) in the calibration set and validation set.

Data Set	PC1	PC2	PC3	PC4	PC5
Calibration set	–0.37	0.31	–0.06	0.43	–0.19
Validation set	–0.08	0.27	–0.42	0.31	–0.05

**Table 3:** Quantitative relationship between SOM and spectral variables of different numbers of dimensions in the calibration (*n* = 112) and validation (*n* = 56) datasets.

Dimensions	Spectral Variables	Linear Model	*R* ^2^_c_	RMSE_c_ (g kg^−1^)	*R* ^2^_v_	RMSE_v_ (g kg^−1^)	RPIQ
1DV	PC1	y = 24.79 − 0.88x	0.14	16.44	0.01	16.36	0.67
	PC2	y = 8.59 + 3.22x	0.10	11.30	0.07	12.20	0.82
	PC3	y = 8.40 − 1.17x	0.01	11.82	0.18	12.77	0.81
	PC4	y = 4.18 + 13.72x	0.19	10.97	0.09	13.79	0.87
	PC5	y = 7.79 − 11.90x	0.04	11.21	0.01	15.00	0.86
2DI	SI (R_2260_, R_1450_)	y = 27.85 − 25.56x	0.34	11.79	0.34	12.59	1.08
	DI (R_800_, R_790_)	y = 20.03 − 348.35x	0.46	9.19	0.43	9.98	1.03
	PI (R_1490_, R_2340_)	y = 21.08 − 86.87x	0.33	9.13	0.30	10.92	1.04
	RI (R_785_, R_805_)	y = −70.94 + 83.80x	0.47	9.58	0.49	10.38	1.10
	NDI (R_800_, R_790_)	y = 19.38 − 353.96x	0.48	9.19	0.46	9.98	1.03
3DI	TBI1 (R_2265_, R_2230_, R_1465_)	y = −83.16 + 174.51x	0.69	6.95	0.64	7.65	1.34
	TBI2 (R_2215_, R_1460_, R_2255_)	y = 128.19 − 62.75x	0.71	6.14	0.70	6.86	1.50
	TBI3 (R_1890_, R_2065_, R_2265_)	y = −4.32 − 21.12x	0.72	7.79	0.68	8.54	1.23
	TBI4 (R_1895_, R_2095_, R_2295_)	y = 2.21 − 8.06x	0.70	8.19	0.68	8.53	1.23
	TBI5 (R_2200_, R_1455_, R_2280_)	y = 9.28 − 263.26x	0.65	7.22	0.64	8.00	1.29

**Table 4:** Statistical summary of SOM developed from each strategy by using the random forest (RF) algorithm.

Strategy	Number of m*_try_*	Calibration (*n* = 112)		Validation (*n* = 56)		
		*R* ^2^_c_	RMSE_c_ (g kg^−1^)	*R* ^2^_V_	RMSE_v_ (g kg^−1^)	RPIQ
1DV	3	0.44	6.35	0.43	7.08	1.45
2DI	5	0.74	4.39	0.70	5.23	1.97
3DI	1	0.89	2.85	0.90	3.27	3.15
1DV+2DI	10	0.83	3.71	0.82	4.16	2.48
1DV+3DI	8	0.91	2.86	0.90	2.96	3.48
2DI+3DI	8	0.94	2.29	0.93	2.52	4.09
